# Antioxidant and Anti-Inflammatory Activity of *Citrus* Flavanones Mix and Its Stability after In Vitro Simulated Digestion

**DOI:** 10.3390/antiox10020140

**Published:** 2021-01-20

**Authors:** Marcella Denaro, Antonella Smeriglio, Domenico Trombetta

**Affiliations:** Department of Chemical, Biological, Pharmaceutical and Environmental Sciences, University of Messina, Viale Palatucci, 98168 Messina, Italy; marcella.denaro@unime.it (M.D.); domenico.trombetta@unime.it (D.T.)

**Keywords:** *Citrus* flavanones, neoeriocitrin, eriocitrin, hesperetin, hesperidin, neohesperidin, antioxidant, anti-inflammatory, in vitro-simulated digestion, Caco-2 cells

## Abstract

Recently, several studies have highlighted the role of *Citrus* flavanones in counteracting oxidative stress and inflammatory response in bowel diseases. The aim of study was to identify the most promising *Citrus* flavanones by a preliminary antioxidant and anti-inflammatory screening by in vitro cell-free assays, and then to mix the most powerful ones in equimolar ratio in order to investigate a potential synergistic activity. The obtained flavanones mix (FM) was then subjected to in vitro simulated digestion to evaluate the availability of the parent compounds at the intestinal level. Finally, the anti-inflammatory activity was investigated on a Caco-2 cell-based model stimulated with interleukin (IL)-1β. FM showed stronger antioxidant and anti-inflammatory activity with respect to the single flavanones, demonstrating the occurrence of synergistic activity. The LC-DAD-ESI-MS/MS analysis of gastric and duodenal digested FM (DFM) showed that all compounds remained unchanged at the end of digestion. As proof, a superimposable behavior was observed between FM and DFM in the anti-inflammatory assay carried out on Caco-2 cells. Indeed, it was observed that both FM and DFM decreased the IL-6, IL-8, and nitric oxide (NO) release similarly to the reference anti-inflammatory drug dexamethasone.

## 1. Introduction

Flavonoids represent one of the most representative classes of plant secondary metabolites, exceeding 8000 compounds, including aglycons, glycosides, and polymers [1] with flavanones, which represents one of the most diffused sub-classes in the *Citrus* genus [2].

They show unquestionable antioxidant and free-radical scavenging, as well as anti-inflammatory properties [3,4,5,6]. Thus, they are considered very promising multi-target agents against a wide range of chronic disorders, such as cardiovascular and intestinal bowel diseases, diabetes, and cancer [7,8,9,10,11,12,13]. Recently, several studies have focused their attention on *Citrus* flavanones and their respective glycosylated derivatives as anti-inflammatory agents, particularly in the context of inflammatory bowel diseases (IBDs). IBDs are characterized by chronic and uncontrolled pro-inflammatory states associated with deregulation of both adaptive and innate immunity of the gastrointestinal tract. As a result, an increase of the vascular permeability and blood flow, as well as an increase of leukocyte mobilization and production of inflammatory mediators occur [14]. The exact cause of these pathologies is not fully known, although it is well-known that several factors, such as immune system disturbance, genetic predisposition, and environmental factors play a predominant role [15]. To date, a resolutive pharmacological treatment is not available and therapeutic strategies are mainly focused on non-specific immunosuppressive drugs [14]. Considering the occurrence and prevalence of these pathologies are destined to grow progressively over the years, research is focused on the study of alternative strategies, which may also lead to the onset of fewer side effects [9,14]. Based on epidemiological studies, diets rich in flavonoids are directly correlated with increased longevity and decreased incidence of intestinal chronic diseases, which can potentially evolve in colorectal cancer [16]. In addition to the direct antioxidant activity as free-radical scavenging or protein inhibition agents, flavonoids are able to activate several antioxidant and protective genes via nuclear transcription factors by inhibiting inflammatory pathways [14]. Moreover, they influence the microbiota composition, favoring the growth of bifidum and lactobacilli bacteria, whose depletion is associated with the inflammatory state [14]. Many studies are currently available on *Citrus* flavanones, *Citrus* fruit, or juice extracts, which highlight the pivotal role of these flavonoids in counteracting oxidative stress and the inflammatory response in murine models of IBD [17,18,19,20,21]. However, to date, no study has ever dealt with a thorough screening of the antioxidant and anti-inflammatory activity of the most representative flavanones of the *Citrus* genus. Indeed, with the exception of naringenin and hesperidin, the other flavanones are either hardly investigated or neglected. Moreover, another often-overlooked aspect is the bioaccessibility of these compounds. In fact, in order to reach the small intestine, where they can carry out their local anti-inflammatory activity, and then be partially absorbed at a systemic level, they are subjected to a whole series of complex mechanisms that involve their transport through the gastrointestinal tract and digestion [22].

Considering this, the aim of our study was, first, to select the most representative flavanones of the *Citrus* genus by performing an in vitro antioxidant and anti-inflammatory screening by means of different in vitro cell-free tests. Subsequently, the most promising ones were mixed in equimolar ratio to obtain a flavanones mix (FM) that was subjected to the same tests to evaluate the occurrence of synergistic effects.

Finally, to evaluate the availability of the FM and its anti-inflammatory effects at the intestinal level, in vitro simulated gastrointestinal digestion was carried out and the digesta were then applied on a Caco-2 cells-based model stimulated with interleukin (IL)-1β, a well-known pro-inflammatory agent.

## 2. Materials and Methods

### 2.1. Chemicals

Neoeriocitrin (eriodictyol-7-*O*-neohesperidoside), eriocitrin (eriodictyol-7-*O*-rutinoside), hesperetin (3′,5,7-trihydroxy-4′-methoxyflavanone), hesperidin (hesperetin-7-*O*-rutinoside), neohesperidin (hesperetin-7-*O*-neohesperidoside), diosmin (diosmetin-7-*O*-rutinoside), neodiosmin (diosmetin-7-*O*-neohesperidoside), naringin (naringenin-7-*O*-neohesperidoside) and tangeretin (4′,5,6,7,8-pentamethoxyflavone) analytical standards (HPLC grade, purity ≥ 98%) were purchased by Extrasynthese (Genay, France). Sodium chloride (NaCl), potassium chloride (KCl), calcium chloride (CaCl_2_), urea, cholesterol, sodium phosphate monobasic (NaH_2_PO_4_), zinc sulfate heptahydrate (ZnSO_4_·7H_2_O), α-amylase from human saliva type XI (A1031-1KU), egg-phosphatidylcholine (PC, 840051P), pepsin from porcine gastric mucosa (P6887), α-chymotrypsin type II from bovine pancreas (C4129), trypsin type IX-S from porcine pancreas (T0303), lipase type VI-S from porcine pancreas (L0382), colipase from porcine pancreas (C3028), α-amylase type VI-B from porcine pancreas, sodium glycodeoxycholate (G9910), taurocholic acid sodium salt hydrate (T4009) as well as analytical and HPLC-grade solvents were purchased from Merck KGaA (Darmstadt, Germany). The gastric lipase was of fungal origin (F-AP15) and it was kindly provided by Amano Enzyme Inc. (Nagoya, Japan). Dulbecco’s Modified Eagle medium and 3-(4,5-dimethylthiazol-2-yl)-2,5-diphenyltetrazolium bromide (MTT) were purchased by VWR (Milan, Italy). All chemicals and reagents of analytical grade used for antioxidant and anti-inflammatory tests were purchased from Sigma-Aldrich (Milan, Italy).

### 2.2. In Vitro Cell-Free Antioxidant and Anti-Inflammatory Screening

In order to select the most promising flavanones, a preliminary antioxidant and anti-inflammatory screening by colorimetric in vitro cell-free assays was carried out on the most representative flavanones in the *Citrus* genus (see Section 2.1). Unless otherwise specified, the absorbance data were recorded by an UV-VIS Spectrophotometer (Shimadzu UV-1601, Kyoto, Japan). Results were expressed as half-maximal inhibitory concentration (IC_50_, µg/mL) with confident limits (C.L.) at 95% calculated by Litchfield and Wilcoxon test, using the PHARM/PCS software version 4 (MCS Consulting, Wynnewood, PA, USA). Data represent the mean ± standard deviation (S.D.) of three independent experiments in triplicate (*n* = 3). The concentration ranges reported below refer to the final concentrations of the samples or reference compounds in the reaction mixture.

#### 2.2.1. Antioxidant Assays

##### Ferric Reducing Antioxidant Power (FRAP) Assay

The FRAP assay was carried out according to Smeriglio et al. [23]. Fifty microliters of each flavanone (3.13–3200 µM) or trolox as reference compound (5.0–40 µM) were added to 1.5 mL of fresh working FRAP reagent consisting of 300 mM acetate buffer (pH 3.6), 10 mM 2,4,6-tris(2-pyridyl)-S-triazine (TPTZ) solution in 40 mM HCl and 20 mM FeCl_3_·6H_2_O solution (10:1:1 *v*/*v*/*v*), pre-warmed at 37 °C. The reaction mixture was incubated for 4 min at room temperature (RT) and the absorbance recorded at 593 nm.

##### Oxygen Radical Absorbance Capacity (ORAC) Assay

The ORAC assay was performed according to Muscarà et al. [24]. Briefly, 20 µL of each flavanone (0.08–800 µM) or trolox as reference compound (1.0–10 µM) were added to fresh 117 nM fluorescein solution (120 µL) diluted in 75 mM phosphate buffer (pH 7.4). After a pre-incubation at 37 °C for 15 min, 60 µL of 40 mM 2,2′-Azobis(2-amidinopropane) dihydrochloride (AAPH radical) solution was added to each well to trigger the reaction, which was monitored every 30 s for 90 min (λ_ex_ 485; λ_em_ 520) by a fluorescence plate reader (FLUOstar Omega, BMG LABTECH, Ortenberg, Germany).

##### Trolox Equivalent Antioxidant Capacity (TEAC) Assay

TEAC assay was carried out according to Bazzicalupo et al. [25]. The reaction mixture, consisting of 4.3 mM potassium persulfate and 1.7 mM 2,2′-azino-bis (3-ethylbenzthiazoline-6-sulfonic acid) (ABTS) 1:5 *v*/*v*, was incubated for 12–16 h in the dark at RT and diluted in deionized water until an absorbance value of 0.7 ± 0.02 at 734 nm. Fifty microliters of each flavanone (2.0–64 µM) or trolox as reference compound (2.50–20 µM) were added to 1 mL of the TEAC reagent and incubated at RT in the dark for 6 min. The absorbance was recorded at 734 nm.

##### DPPH (2,2-Difenil-1-Picrylhydrazyl) Assay

The radical scavenging activity of samples against DPPH was evaluated according to Smeriglio et al. [26]. Briefly, 37.5 µL of each flavanone (3.13–6400 µM) or trolox as reference compound (2.5–20 µM) were added to 1.5 mL of fresh 10^−4^ M DPPH methanol solution, mixed for 10 s and incubated for 20 min in the dark at RT. The absorbance was recorded at 517 nm.

#### 2.2.2. Anti-Inflammatory Assays

##### Albumin Denaturation Assay (ADA)

This assay evaluates the inhibitory activity of sample on heat-induced denaturation of bovine serum albumin (BSA) [27]. Briefly, 80 µL of each flavanone (100–6400 µM) or diclofenac sodium as reference compound (4.0–32 µM) were added to 100 µL of 0.4% fatty free BSA solution and 20 µL of phosphate buffer saline (PBS, pH 5.3) into a 96 well-plate. The absorbance was recorded at 595 nm at the starting time (t = 0) and after 30 min (t = 30) at 70 °C by a microplate reader (Multiskan GO, Thermo Scientific, Waltham, MA, USA). A blank consisting of PBS instead of sample was used as negative control. The inhibition percentage of denaturation (ID %) was calculated as follows:

% ID = ((1 − (A − B))/(C − B)) × 100(1)

where A = sample absorbance (*t* = 30); B = blank absorbance at (*t* = 0); C = blank absorbance at (*t* = 30).

##### Anti-Protease Activity (APA)

The anti-tryptic activity of sample was evaluated according to Smeriglio et al. [27]. Briefly, 200 µL of each flavanone (6.25–140 µM) or diclofenac sodium as reference compound (10–80 µM) were added to 12 µL of trypsin (10 µg/mL) and 188 µL of 25 mM Tris-HCl buffer (pH 7.5). After that, 200 µL of 0.8% casein solution were added and samples incubated for 20 min at 37 °C in a water bath. After that, 400 µL of perchloric acid were added to stop the reaction. The cloudy suspension, due to the protein precipitation, was centrifuged at 3500× *g* for 10 min and the absorbance of the supernatant was recorded at 280 nm against a blank consisting of deionized water instead of sample.

### 2.3. Flavanones Mix Preparation

Based on the results of the preliminary antioxidant and anti-inflammatory screening (see Section 2.2), the most powerful flavanones (neohesperidin, hesperidin, neoeriocitrin, eriocitrin, and hesperetin) were selected in order to prepare a flavanones mix (FM), which was, at first instance, investigated by the same in vitro colorimetric assays (see Section 2.2) in order to evaluate its potency with respect to the single flavanones and to elucidate potential synergistic mechanisms. The following final concentration range of FM were used: FRAP (2.50–20 µM), ORAC (0.08–0.60 µM), TEAC (0.31–2.50 µM), DPPH (1.25–10 µM), ADA (5.0–40 µM), and APA (2.50–20 µM). After that, being the results of the preliminary antioxidant and anti-inflammatory screening of FM very promising, an appropriate concentration was chosen to proceed with the subsequent analyses. In particular, the FM was prepared on the assumption that digested sample, which would then be applied to the Caco-2 cells for the anti-inflammatory experiments, had to contain an equimolar concentration of the five most powerful flavanones (neohesperidin, hesperidin, neoeriocitrin, eriocitrin, and hesperetin) equal to 10 µM, which represents the mean efficacy concentration taking into account the IC_50_ values obtained in the preliminary antioxidant and anti-inflammatory screening of the FM. At this purpose, stock solutions (14 mM) of flavanones in DMSO were mixed and diluted 10-fold with Milli-Q water to obtain the 1.4 mM FM, which was used to carried out the in vitro simulated human digestion.

### 2.4. In Vitro Simulated Human Digestion

In vitro gastric and duodenal digestion of FM was carried out according to Trombetta et al. [28] with some modifications. FM solution (1.5 mL) (see Section 2.3), has been dissolved in 7.5 mL of simulated gastric solution containing 0.127 mM egg-phosphatidylcholine and adjusting the pH to 2.5, by 1 M HCl. Gastric pepsin and lipase (9000 U/mL and 60 U/mL, respectively) were added to the mixture starting the gastric digestion phase, which was protracted for 2 h at 37 °C, incubating under continuous stirring (170 rpm) by an Innova 4000 Benchtop Incubator Shaker (New Brunswick Scientific, Edison, NJ, USA). The gastric phase was stopped by the addition of 1M NaOH in order to reach a pH of 7.5, and 6 mL of the previous solution passed to the duodenal digestion phase. Duodenal solution was prepared by adding 2.10 mL of simulated bile solution (6.5 mM phosphatidylcholine, 4 mM cholesterol, 12.5 mM sodium taurocholate, and 12.5 mM sodium glycodeoxycholate) to 5.9 mL of simulated pancreatic juice containing pancreatic lipase (590 U/mL), colipase (3.2 µg/mL), trypsin (11 U/mL), α-chymotrypsin (24 U/mL), and α-amylase (300 U/mL). The duodenal mixture was incubated for 4 h under continuous stirring at 37 °C as described above. At the end, both gastric and duodenal digesta were immediately stored at −80 °C until the subsequent analyses.

### 2.5. Pre- and Post-Digestion Analyses

For quali-quantitative determination of flavanones, gastric and duodenal digesta were extracted with methanol (1:2, *v*/*v*) by mixing for 3 min. After that, samples were centrifuged at 12,000× *g* for 10 min, filtered by a 0.22 µm nylon syringe filter and injected into a liquid chromatograph equipped with a diode-array detector and mass spectrophotometer (LC-DAD-MS system, Agilent technologies, Santa Clara, CA, USA). The chromatographic separation was obtained using a reverse phase column Luna Omega PS C18 (150 × 2.1 mm, 5 µm; Phenomenex, Torrance, CA, USA) by using solvent A (formic acid 0.1% *v/v*) and solvent B (acetonitrile) as mobile phase according to the following elution gradient: 0 min, 0% B; 3–9 min, 3% B; 9–24 min, 12% B; 24–30 min, 20% B; 30–33 min, 33–43 30% B; 43–66 min 50% di B; 66–81 min 60% di B; 81–86 min 0% di B; 86–90 min 0% di B. Flow rate was 0.4 mL/min and injection volume and column temperature were set at 5 μL and 25 °C, respectively. UV–Vis spectra of flavanones were recorded (190–400 nm) and chromatograms were acquired at 292 nm, maximum flavanones absorption wavelength.

For MS analysis, an ion trap fitted with an electrospray ionization (ESI) source operating in negative ionization mode, was used. Nitrogen (dry gas) flow rate and pressure were set at 9 L/min and 40 psi, respectively, whereas the nebulizer temperature was set at 350 °C. Helium was used as collision gas (1.46 × 10^−5^ bar) and collision-induced dissociation spectra were obtained with a fragmentation amplitude of 1.0 V (MS/MS). Data acquisition was performed using the ChemStation A.10.01 software (Agilent technologies, Santa Clara, CA, USA). The identification of flavanones, as well as of any metabolites, was carried out by comparison of retention time, UV-Vis and mass spectra of the analytes with reference compounds where possible or by comparison of UV-VIS and mass spectra with data reported in literature and mass spectra libraries. Quantification was carried out by built external standard calibration curves of each flavanone (0.612–10 µg/mL) and expressing the results as average ± standard deviation (S.D.) of three independent experiments in triplicate (*n* = 3).

The HPLC method was validated according to the current international guidelines [29], regarding selectivity, linearity, precision, robustness, limit of detection (LOD), limit of quantitation (LOQ) and recovery. LOD and LOQ values were calculated following the approach based on the standard deviation of the response and the slope of the calibration curves [29].

### 2.6. Anti-Inflammatory Activity on In Vitro Cell-Based Model

#### 2.6.1. Cell Model

Experiments were carried out on transwell models (CacoReady™, Readycell, Barcelona, Spain) consisting of Caco-2 cells (8.5 × 104 cells/cm^2^, passage number 41–58) seeded on polyester microporous filters in 24-well HTS plates (6.5 mm diameter, 0.33 cm^2^ area and 0.4 µm of pore size) (Corning Incorporated, Corning, NY, USA). Completed Dulbecco’s Modified Eagle Medium (DMEM), prepared according to Denaro et al. [30], was added on the apical (0.3 mL) and basolateral side (0.9 mL) of the Transwell^®^ insert. After 21 days of culture, Caco-2 cells were completely differentiated and polarized, such that their phenotype resembled the morphological and functional features of mature enterocytes lining the small intestine.

#### 2.6.2. Anti-Inflammatory Assay

Before starting with the anti-inflammatory experiments, the monolayer integrity was checked by measuring the trans-epithelial electrical resistance (TEER) with a Millicell^®^ ERS-2 V/ohmmeter (Merck Millipore, Darmstadt, Germany) equipped with STX 100C electrode (World Precision Instruments, Sarasota, FL, USA). Caco-2-plated filters with epithelial resistance ≥800 Ω/cm^2^ were used.

The anti-inflammatory activity of FM and digested FM (DFM) was evaluated on Caco-2 transwell model, according to Tesoriere et al. [31], with some modifications. Briefly, 1.4 mM FM (see Section 2.3) was diluted 140 times in completed DMEM, obtaining a 10 µM FM (DMSO < 0.1%). DFM samples, which had at the end of digestion a concentration equal to 100 µM, were diluted 10 times in completed DMEM obtaining a 10 µM solution (DMSO < 0.1%). To trigger inflammation, 25 ng/mL IL-1β was used.

Cell monolayers were treated on the apical side with 0.25 mL of 10 µM FM, 10 µM DFM, 10 µM dexamethasone (Dex) (reference anti-inflammatory drug), 10 µM FM/IL-1β, 10 µM DFM/IL-1β and 10 µM Dex/IL-1β. DMEM containing 0.1% DMSO and 25 ng/mL IL-1β were used as negative and positive control, respectively. Completed DMEM (0.75 mL) was added on the basolateral side and cells were incubated for 24 h at 37 °C, 5% CO_2_. Three independent experiments in triplicate (*n* = 3) were carried out for each cell treatment.

Samples were collected and stored at −80 °C until the subsequent analyses. Post-quality control assays, such as TEER, as well as the apparent permeability coefficient (Papp) and paracellular flux (Pf) of Lucifer yellow (LY) were assessed in order to evaluate the Caco-2 monolayer integrity [30].

#### 2.6.3. Determination of Inflammatory Markers

IL-6 and IL-8 release were measured by high-sensitivity human ELISA kits (Cymax IL-6 YIF-LF-EK0260 and Cymax IL-8YIF-LF-EK0262, Ab Frontiers, Adipogen Corporation, 9853 Pacific Heights Blvd., Suite L San Diego, CA, USA) according to the manufacturer’s recommendations, whereas NO release was estimated according to Tesoriere et al. [31] by Griess reaction. Briefly, 100 µL of sample were mixed with 100 µL of Griess reagent (1% sulfanilamide (*w*/*v*) in 5% phosphoric acid (*v*/*v*) and 0.1% of naphthylethylenediamine-HCl (*w*/*v*), 1:1 *v*/*v*), and incubated for 10 min at RT. Absorbance was recorded at 550 nm using a microplate reader (Multiskan GO, Thermo Scientific, Waltham, MA, USA). Results were expressed as µM NO extrapolating the results by a reference standard curve of sodium nitrite (1.0–15 µM).

#### 2.6.4. Cell Viability

Cell viability was assessed by 3-(4,5-dimethylthiazol-2-yl)-2,5-diphenyltetrazolium bromide (MTT) assay according to Kenzaoui and co-workers [32].

### 2.7. Statistical Analysis

Three independent experiments in triplicate (*n* = 3) were carried out for each in vitro cell-free and cell-based assay. Results were expressed as mean ± standard deviation (S.D.). Data were analyzed by one-way analysis of variance (ANOVA) followed by Tukey’s test by SigmaPlot12 (Systat Software, Inc., San Jose, CA, USA). Results were statistically significant for *p* ≤ 0.05.

## 3. Results

### 3.1. Antioxidant and Anti-Inflammatory Screening of Flavanones and Flavanones Mix

**Figure 1:**
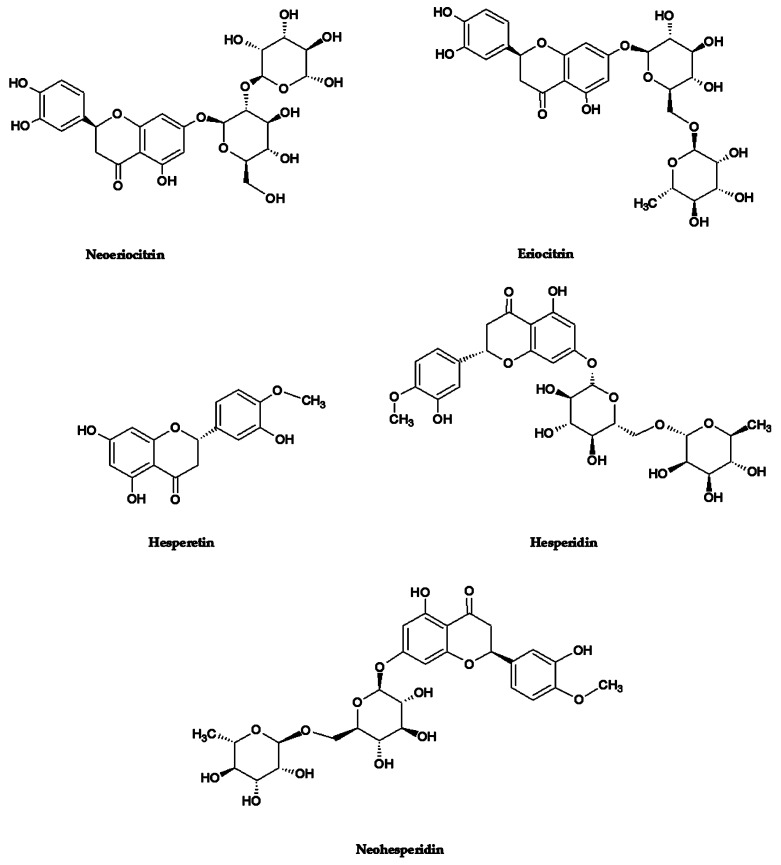
Chemical structures of the selected most powerful *Citrus* flavanones.

With the aim of identifying the most promising flavanones from an antioxidant and anti-inflammatory point of view, nine of the most representative compounds of the *Citrus* genus were chosen: neoeriocitrin, eriocitrin, hesperetin, hesperidin, neohesperidin, diosmin, neodiosmin, naringin, and tangeretin. Among these, after a preliminary antioxidant and anti-inflammatory screening by in vitro cell-free assays, the five most promising ones were selected (Figure 1) and suitably mixed in equimolar ratio (flavanones mix) in order to elucidate possible synergistic effects.

**Figure 2:**
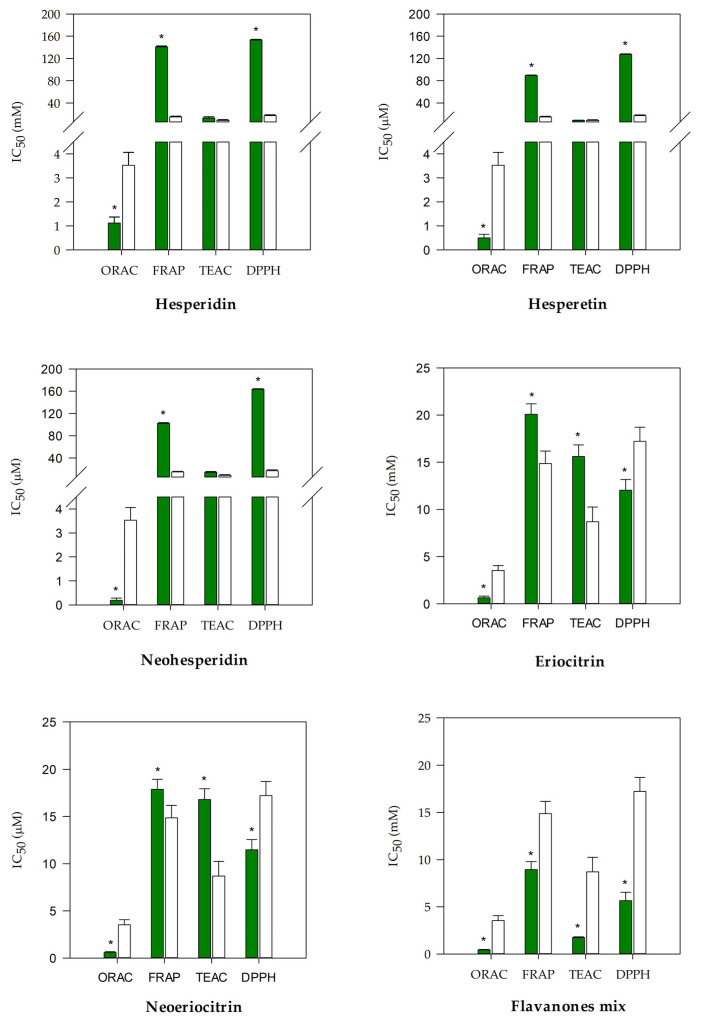
Antioxidant and free radical-scavenging activity of *Citrus* flavanones (**green bars**) in comparison with the reference compound trolox (**white bars**). Results were expressed as mean half-maximal inhibitory concentration (IC_50_, µM) ± S.D. of three independent experiments in triplicate (*n* = 3); * *p* < 0.001 vs. trolox.

The antioxidant screening was carried out using four different colorimetric tests (ORAC, FRAP, TEAC, and DPPH) characterized by different environments and reaction mechanisms, as well as by different charged radicals. Results were depicted in Figure 2, which reports the IC_50_ of the five strongest flavanones (neohesperidin, hesperidin, neoeriocitrin, eriocitrin, and hesperetin) in comparison with the reference standard trolox.

All investigated flavanones showed the same behavior, showing a greater antioxidant activity in the ORAC test followed by TEAC, FRAP, and DPPH, with the exception of eriocitrin and neoeriocitrin, which showed instead the following order of potency: ORAC > DPPH > TEAC > FRAP. These results shed light on the importance of studying the antioxidant activity with different tests, because the response of a molecule can vary substantially depending on its structural features. Among the strongest molecules, surely stood out hesperidin, hesperetin, and neohesperidin in the TEAC and ORAC tests, and eriocitrin and neoeriocitrin in the FRAP and DPPH tests, which showed different behavior, depending on the radical charge and test reaction mechanism (based on transfer of hydrogen atoms for TEAC and ORAC, and on electron transfer for FRAP and DPPH). The other flavanones taking into account at the beginning of study, were discarded because they were less active with respect to the five selected flavanones, with IC_50_ values ≥2 times in ORAC and TEAC assays and IC_50_ ≥ 4 times in FRAP and DPPH assays (data not shown). The antioxidant activity of the five selected flavanones resulted statistically significant (*p* < 0.001) with respect to the trolox in all of the tests carried out, with the exception of TEAC, in which hesperidin, hesperetin and neohesperidin showed a behavior comparable with the reference standard (Figure 2). Furthermore, it is interesting to note that all five tested flavanones were significantly (*p* < 0.001) stronger with respect to the trolox in the ORAC assay.

In the last panel of Figure 2, it is possible to observe the behavior of the flavanones mix (FM). By averaging the IC_50_ value of the five flavanones in each test, it is easy to see how the FM always showed the greater antioxidant activity (17 times stronger in the DPPH, 8.6 times stronger in the FRAP, 7.8 times stronger in the TEAC, and 1.4 times stronger in the ORAC) compared to the single flavanones, demonstrating that there is effectively a synergistic activity between these molecules, which enhances their intrinsic antioxidant activity. The IC_50_ of the FM is, in fact, always statistically significant (*p* < 0.001) in comparison with that of flavanones in all of the tests carried out (*p* < 0.001, data not shown). Moreover, an even more interesting aspect is that the FM IC_50_ was always significantly lower (*p* < 0.001) in comparison with the reference standard (trolox).

**Figure 3:**
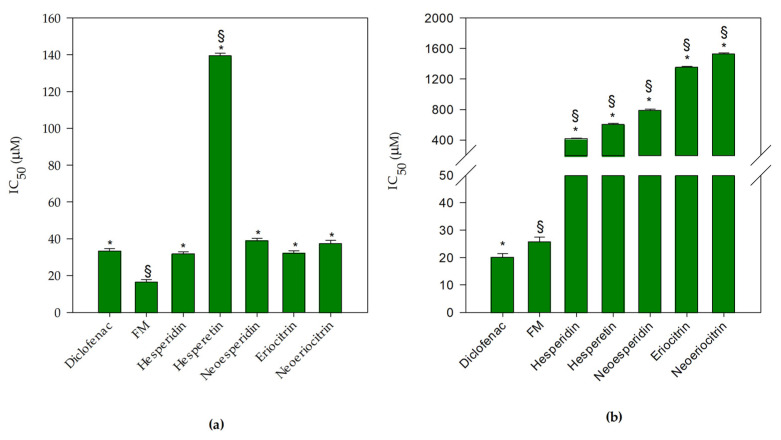
Anti-inflammatory activity of *Citrus* flavanones and flavanones mix (FM) in comparison with the reference anti-inflammatory drug diclofenac sodium: (**a**) protease inhibition assay (**b**) bovine serum albumin (BSA) denaturation assay. Results were expressed as mean half-maximal inhibitory concentration (IC_50_, µM) ± S.D. of three independent experiments in triplicate (*n* = 3); * *p* < 0.001 vs. FM; ^§^ *p* < 0.001 vs. diclofenac sodium.

These preliminary results were corroborated by the evaluation of the anti-inflammatory activity, investigated by the protease inhibition assay and the heat-induced denaturation of BSA. In Figure 3a, it is possible to observe and compare the IC_50_ of each flavanone and FM, with respect to the reference anti-inflammatory drug diclofenac sodium in the protease inhibition test.

FM and hesperetin showed a statistically significant activity (*p* < 0.001) in comparison with diclofenac sodium. In particular, FM showed the strongest anti-inflammatory activity (about 2 times greater with respect to the reference compound), whereas hesperetin showed the lowest anti-inflammatory activity (about 4 times less) than the other tested molecules, which instead showed a comparable behavior, with respect to the reference standard. In regards to the inhibition of the BSA denaturation, FM showed a significantly higher activity (*p* < 0.001) in comparison with the single flavanones (about 38 times stronger, considering an average of the IC_50_ of the single compounds) and a significantly lower activity with respect to the diclofenac sodium (approximately 1.3 times).

Therefore, in accordance with the previous results, it is possible to postulate a clear synergistic mechanism of FM, capable of significantly enhancing the intrinsic anti-inflammatory activity of the individual molecules.

### 3.2. Pre- and Post-Digestion Analysis of Flavanones Mix

The quali-quantitative determination of flavanones in FM and gastric and duodenal DFM, was carried out by a LC-DAD-ESI-MS/MS analysis. The analytical method was validated and, as observed from the data, reported in Table 1, the method resulted as sensitive, precise, accurate, and repeatable, with recovery values ≥90.36%. Moreover, according to the current international guidelines [29], the robustness of the method was evaluated, taking into account the following parameters: pH variation of mobile phase, variations in mobile phase composition, different lots, and/or suppliers of column, temperature, and flow rate. Maintaining the conditions reported in Section 2.5, the analytical method results robust, because it is reliable, with respect to deliberate variations in the method parameters mentioned above (data not shown).

**Figure 4:**
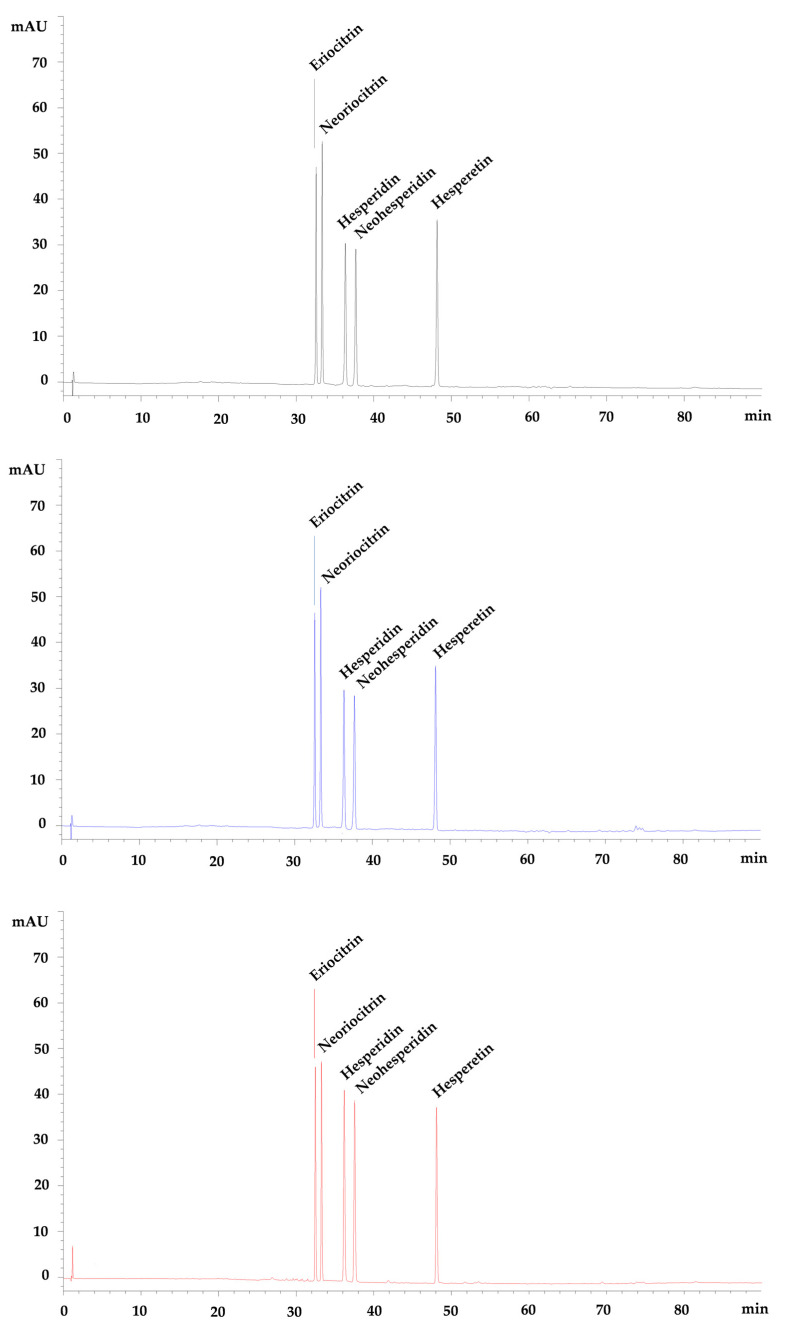
Representative LC-DAD chromatograms of flavanones mix (**black**), gastric digested flavanones mix (**blue**) and duodenal digested flavanones mix (**red**) acquired at 292 nm.

Representative chromatograms acquired at 292 nm of the FM (black), as well as of the gastric (blue) and duodenal (red) DFM, are reported in Figure 4.

No interferences, such as degradation products, metabolites, or co-eluting compounds, were recorded. Moreover, the chromatographic separation of the constituents of the FM did not show any overlap or interferences from matrix constituents in the digested samples at the retention time of the five flavanones, which appeared well-separated and easy identifiable. No metabolites were identified in the gastric digested samples or in the duodenal digested samples, as observed in Figure 4 and Table 2, in which the five flavanones are identified and quantified, with respect to the starting FM (10 µM).

Indeed, only a mild decrease ≤10.8% in gastric digested samples followed by a further decrease ≤1.6% in duodenal digested samples was observed, recording a total flavanones loss ≤12.4%. This loss is certainly attributable to the sample extraction process, as it is possible to observe from the recovery data reported in Table 1.

### 3.3. Anti-Inflammatory Activity of Flavanones Mix on Caco-2 Cells

Once FM was identified as the most promising formulation from an antioxidant and anti-inflammatory point of view, an anti-inflammatory activity study was conducted on a Caco-2 cell-based model, to evaluate the potential applicability of this FM as an anti-inflammatory agent, and plan further (and more in-depth) in vivo studies on animal models.

**Figure 5:**
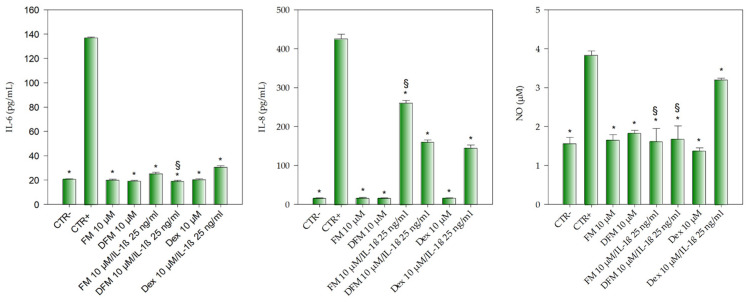
Evaluation of the anti-inflammatory activity of flavanones mix (FM, 10 µM) and gastric plus duodenal digested flavanones mix (DFM, 10 µM) in comparison with the reference anti-inflammatory drug dexamethasone (Dex, 10 µM) by a cell-based assay carried out on Caco-2 transwell model. IL-1β (25 ng/mL) was used to trigger the inflammation (CTR^+^), whereas DMEM with 0.1% DMSO was used as negative control (CTR^−^). Results were expressed as the concentration (pg/mL for IL-6 and IL-8, and µM for NO) of inflammatory markers released after cell treatments ± S.D. of three independent experiments in triplicate (*n* = 3); * *p* < 0.001 vs. CTR^+^; ^§^ *p* < 0.001 vs. Dex 10 µM/IL-1β 25 ng/mL.

As can be seen from Figure 5, the inflammation triggered by 25 ng/mL IL-1β allowed a statistically significant (*p* < 0.001) increase in IL-6 (~7 folds vs. CTR^−^), IL-8 (~27 folds vs. CTR^−^) as well as in NO release (~2.5 folds vs. CTR^−^), well-known inflammatory mediators.

FM, as well as the digested flavanones mix (DFM) and dexamethasone (Dex), all at the same concentration (10 µM), did not show any pro-inflammatory activity, so much so that they did not show any statistically significant difference between them, and between them and CTR^−^, for all the three inflammatory markers taking into account.

The co-treatment of Caco-2 cells with IL-1β 25 ng/mL and 10 µM FM, 10 µM DFM and 10 µM Dex, showed a statistically significant decrease (*p* < 0.001) of IL-6 release with respect to the positive control (CTR^+^, 25 ng/mL IL-1β). Moreover, what appears even more interesting is that the DFM 10 µM/IL-1β co-treatment showed a statistically significant decrease (*p* < 0.001) in IL-6 release even compared to the Dex co-treatment (Dex 10 µM/IL-1β) (Figure 5).

A different behavior was instead observed with regard the IL-8 and NO release (Figure 5). Indeed, in the first case, a statistically significant difference (*p* < 0.001) in the inhibition of IL-8 release was found between the FM co-treatment (FM 10 µM/IL-1β) and the Dex co-treatment (Dex 10 µM/IL-1β) (~39 and 66%, respectively), whereas the DFM co-treatment (DFM 10 µM/IL-1β) showed a superimposable behavior to that of the Dex co-treatment (Dex 10 µM/IL-1β) inhibiting the IL-8 release by ~62%. Finally, both the FM 10 µM/IL-1βand the DFM 10 µM/IL-1β co-treatments showed an inhibitory activity on NO release (~58% and 56%, respectively), significantly higher (*p* < 0.001) with respect to the Dex 10 µM/IL-1β co-treatment, which showed approximately an inhibition of 16%.

No cytotoxicity or alteration of the barrier systems was observed after anti-inflammatory assays. Cell viability results did not show any statistically significant difference in respect to negative control (cell viability ≥ 95.62% ± 4.24), and TEER (800–1000 Ω/cm^2^), LY Papp and Pf (≤9.25 × 10^−7^ cm/s and ≤0.33%, respectively) were always within the reference standard values (200–1000 Ω/cm^2^, LY Papp (cm/s) ≤ 2 × 10^−6^ and Pf ≤ 0.7%) [33,34].

## 4. Discussion

The bioavailability of flavanones has not been fully clarified, yet several studies have focused on the metabolic patterns of these compounds, trying to understand if the observed biological activities are due, at least in part, to the parent compounds. Evidence suggests that they may be metabolized by the intestinal microbiome, resulting in the formation of aglycones and smaller phenolics, which affect the bioavailability of the parent compounds [10]. However, what emerges from the literature is the lack of studies that evaluate the bioaccessibility of these compounds, which is the fraction of compounds available for the absorption in the small intestine. This aspect becomes even more important when it is necessary to evaluate the anti-inflammatory activity of a compound or formulation at intestinal level. Indeed, since these compounds must exert their anti-inflammatory activity locally, it is important to know what is the real availability of these compounds in the intestinal compartment after the digestive processes. Despite several studies investigating the antioxidant and anti-inflammatory activity of some *Citrus* flavanones, such as hesperidin, hesperetin, and naringenin [35,36,37,38] are available, this is the first one that carried out an antioxidant and anti-inflammatory screening of the most representative flavanones of the *Citrus* genus. Moreover, for the first time, the most promising molecules have been selected and mixed in equimolar ratio to investigate the interactions between these compounds and the potential occurrence of synergistic effects, which are often observed with *Citrus* extracts or juices containing different molecules belonging to the same or different classes of flavonoids [26,27], as well as for other medicinal plants, such as *Opuntia ficus indica* L., *Cannabis sativa* L. and *Echinacea purpurea* (L.) Moench [23,39,40].

The investigated FM (hesperetin, hesperidin, neohesperidin, eriocitrin, and neoeriocitrin) was then subjected to simulated digestion in vitro to mimic what happens in vivo, and to verify what occurs to these compounds once ingested along the gastrointestinal tract. The LC-DAD-ESI-MS/MS analysis of gastric and duodenal DFM showed that any compound has been altered during gastric digestion, and this condition remains unchanged in duodenal digestion too.

This stability was previously described in vitro and in vivo for some of the flavanones described in the present study (neohesperidin, hesperidin, and hesperetin) as most representative compounds of a bitter orange extract [41]. Results showed that these flavanones were stable after simulated in vitro digestion. Moreover, they were detected both in the small intestine and in the colon of rats, even 12 and 24 h after oral administration of the bitter orange extract, with a maximum recovery percentage of 72% with respect to the ingested dose [41]. These results corroborate that dietary flavanones can remain unaltered for several hours in the colon where they may be absorbed and/or interact with the intestinal mucosa exerting the local anti-inflammatory activity.

A possible explanation for this particular behavior lies in the peculiar metabolisms of rutinoside and neohesperidoside derivatives, which usually arrive unchanged in the distal part of the intestine, where they are hydrolyzed by colonic microflora enzymes and then absorbed [42]. Considering this, the results obtained in the present study are mainly attributable to the structure of the five selected flavanones: two rutinosides (hesperidin and eriocitrin), two neohesperidosides (neohesperidin and neoeriocitrin), and one aglycon (hesperetin), which not only showed the strongest antioxidant and anti-inflammatory activities, with respect to other *Citrus* flavanones tested, but they have maintained their structure. Consequently, their activities were unchanged, even after being subjected to simulated digestion. In fact, no chemical or enzymatic degradation was observed after gastric and duodenal digestion, indirectly demonstrating how the presence of these sugars shields the molecule from possible enzymatic attacks or hydrolytic phenomena due to pH changes, which happens, on the contrary for glucose conjugates. It has been demonstrated that glucose conjugates, but not conjugates with other sugars, such as rhamnose and rutinose, are rapidly hydrolyzed in the oral cavity for the presence of β-glucosidases, enzymes expressed abundantly at the intestinal level [43].

Several anti-inflammatory studies have been carried out to understand how flavanones modulate inflammatory response. Results obtained from the investigation on IL-1β stimulated Caco-2 cells showed that FM mix and DFM were able to decrease the release of the inflammatory markers IL-6, IL-8, and NO already at “pharma-nutritional” doses. Indeed, the FM and DFM concentration used (10 µM) is a concentration easily reachable even by simply consuming *Citrus* fruits or juices [3]. DFM showed, at times, a stronger anti-inflammatory activity than the FM mix, although the LC-DAD-ESI-MS/MS analysis did not show any presence of metabolites or degradation products. This finding could be related to the presence of several enzymes in DFM, which exert their own anti-inflammatory activity. It has been demonstrated that proteolytic enzymes, such as chymotrypsin and trypsin, possess anti-inflammatory activities, and exhibit synergistic effects with anti-inflammatory drugs [44].

The flavanones ability to modulate inflammation depends on different factors. One of these is a consequence of the antioxidant potency, which is strongly related to their structure, such as presence of sugar moieties, number of hydroxyl groups, and their spatial arrangement [3]. Equally important in inflammatory modulation is the inhibitory activity of these compounds on key enzymes responsible for activation and transduction of inflammatory stimuli; for example, hesperidin was able to inhibit mitogen-activated protein kinases (MAPKs) and phosphodiesterases [45]. The MAPKs role in intracellular signaling pathway during pro-inflammatory response is closely related to the NF-kb signaling pathway, which exerts the pivotal role in the expression of iNOS, COX-2, IL-6, and TNF-α [46]. It was demonstrated that flavanones are able to downregulate NF-Kb [47], but the differences in the experimental models can affect the outcomes, which can appear discordant, and needs to be separately interpreted [46]. Recently, Guazelli et al. [48] corroborated the previous observations by investigation of the antioxidant and anti-inflammatory effects of hesperidin methyl chalcone on an animal model of ulcerative colitis. The authors highlighted, in particular, as this methylated derivative of hesperidin can play a pivotal role in increasing the antioxidant defense system, counteracting the inflammatory state by decreasing the pro-inflammatory cytokines release (TNF-α, IL-6, IL-1β, and IL-33) in the colon via NF-Kb, by improving, significantly, the macro and microscopic damages induced by intracolonic administration of acetic acid in rats.

However, despite the abundance of studies on flavanones, their anti-inflammatory mechanisms of action remain unclear, and further studies are needed to better clarify their roles in modulating the inflammatory cascade.

The results of the present study open new perspectives for the possible clinical use of this FM for inflammatory intestinal diseases, from the mild forms, to the more severe, such as ulcerative colitis and Crohn’s disease, which are becoming increasingly widespread.

The therapeutic strategies available today mainly focus on the use of corticosteroids and immunosuppressive agents, aim to achieve and maintain remission, and prevent disease progression. However, these drugs have several side effects, and some patients quickly become refractory to the treatment (in some cases requiring surgery) [49]. From this point of view, new nutraceutical formulations, which have efficacy equal to synthetic drugs, but with fewer or no side effects, represent the therapeutic strategy of the future, both alone and in co-treatment with conventional drugs. Moreover, it has been demonstrated that naringin, a flavanone widespread in the *Citrus* genus, could be a useful agent to counteract bone loss due to steroid treatment of inflammatory intestinal diseases [50], opening new perspectives in the treatment of adverse effects of conventional drugs.

Despite this being an innovative study, because it investigates for the first time, to our knowledge, the synergistic antioxidant and anti-inflammatory activity of a *Citrus* flavanones mix on in vitro cell-free and cell-based models, and its stability after simulated gastrointestinal digestion, this is a preliminary study, and, furthermore, it shows several limitations. First, only the IL-1β-induced inflammation was used. Second, the in vitro intestinal model used reproduces only partially the intestinal environment lacking the microbiome. Third, we did not investigate the molecular mechanism underlying intestinal inflammatory activity. Considering this, further in vitro cell-based co-culture studies, as well as investigation on specific IBD animal models, are needed to verify the transability of these results on humans, to confirm the postulated clinical applications.

## 5. Conclusions

In conclusion, the present study elucidates (for the first time) that eriocitrin, neoeriocitrin, hesperidin, neohesperidin, and hesperetin are the most powerful antioxidant and anti-inflammatory compounds among the investigated *Citrus* flavanones, and that the particular structural features of these molecules shield them from possible enzymatic attacks or hydrolytic phenomena during simulated digestion, making them available at the intestinal level, where they can exert their remarkable anti-inflammatory activities. Moreover, this study demonstrates (for the first time) that mixing different flavanones enhances the antioxidant and anti-inflammatory activities of individual compounds, experimentally demonstrating a synergistic effect. The flavanones mix exerts already at a pharma-nutritional dose, an anti-inflammatory activity comparable or higher with respect to the reference drugs diclofenac sodium and dexamethasone.

However, this is a preliminary study based on in vitro cell-free and cell-based models; therefore, the translation of results to the complex in vivo scenario is quite difficult. Considering this, further in vitro cell-based studies, as well as animal and human studies, should be performed in order to investigate, in depth, the anti-inflammatory properties of this flavanones mix, as well as the molecular mechanisms and cellular targets involved, which could justify the potential role of this formulation as a possible alternative strategy to counteract the inflammatory intestinal diseases.

## Figures and Tables

**Table 1:** Method validation parameters.

Flavanone	Calibration Range (µM)	Equation	Linearity (R^2^)	R.S.D. ^1^ (%), *n* = 6 Within-Day	R.S.D. (%), *n* = 6 Between-Day	LOD ^2^ (µM)	LOQ ^3^ (µM)	Recovery (%)
Eriocitrin	0.625–20.0	*y* = 19.891*x*	0.9993	0.177	0.177	0.014	0.042	95.28
Neoeriocitrin	0.625–20.0	*y* = 9.627*x*	0.9998	0.240	0.240	0.027	0.081	94.79
Hesperidin	0.625–20.0	*y* = 22.944*x*	0.9999	0.230	0.230	0.016	0.050	99.48
Neohesperidin	0.625–20.0	*y* = 17.954*x*	0.9994	0.275	0.275	0.015	0.045	97.83
Hesperetin	0.625–20.0	*y* = 26.473*x*	0.9998	0.243	0.243	0.049	0.148	90.36

^1^ Relative standard deviation; ^2^ Limit of detection; ^3^ Limit of quantification.

**Table 2:** Quali-quantitative analysis of flavanones mix pre-and post-simulated human digestion.

Flavanone	RT ^1^	λ_max_^2^	MW ^3^	[M−H]^−^ (*m*/*z*)	MS/MS (*m*/*z*)	FM ^4^ (µM)	GFM ^5^ (µM)	DFM ^6^ (µM)
Eriocitrin	32.460	284; 336	596.5	595	459; 287	10	9.05 ± 0.12	8.80 ± 0.03
Neoeriocitrin	33.256	284; 334	596.5	595	459; 287	10	8.92 ± 0.08	8.77 ± 0.16
Hesperidin	36.183	284; 332	610.5	609	301; 286	10	9.95 ± 0.04	9.86 ± 0.13
Neohesperidin	37.520	284; 334	610.5	609	301; 286	10	9.19 ± 0.22	9.04 ± 0.18
Hesperetin	48.077	288; 336	302.2	301	286	10	9.38 ± 0.18	8.76 ± 0.22

^1^ Retention time; ^2^ Maximum absorption wavelengths; ^3^ Molecular weight; ^4^ Flavanones mix; ^5^ Gastric digested flavanones mix; ^6^ Duodenal digested flavanones mix.

## Data Availability

The data presented in this study are available on request from the corresponding author.
